# Nonlinear Optical Saturable Absorption Properties of 2D VP Nanosheets and Application as SA in a Passively Q-Switched Nd:YVO_4_ Laser

**DOI:** 10.3390/ma17112585

**Published:** 2024-05-28

**Authors:** Haowen Guo, Chunyan Jia, Yongping Yao, Meng Bai, Tiejun Ma, Jiayu Zhang, Jinbao Xia, Hongkun Nie, Bo Yao, Jingliang He, Baitao Zhang

**Affiliations:** 1 State Key Laboratory of Crystal Materials, Institute of Novel Semiconductors, Shandong University, Jinan 250100, China; 2 Key Laboratory of Laser & Infrared System, Ministry of Education, Shandong University, Qingdao 266237, China

**Keywords:** 2D VP nanosheets, saturable absorption properties, all-solid-state laser, pulsed laser

## Abstract

Two-dimensional (2D) violet phosphorus (VP) plays a significant role in the applications of photonic and optoelectronic devices due to its unique optical and electrical properties. The ultrafast carrier dynamics and nonlinear optical absorption properties were systematically investigated here. The intra- and inter-band ultrafast relaxation times of 2D VP nanosheets were measured to be ~6.83 ps and ~62.91 ps using the pump–probe method with a probe laser operating at 1.03 μm. The nonlinear absorption coefficient βeff, the saturation intensity Is, the modulation depth ΔR, and the nonsaturable loss were determined to be −2.18 × 10^4^ cm/MW, 329 kW/cm^2^, 6.3%, and 9.8%, respectively, by using the Z-scan and I-scan methods, indicating the tremendous saturable absorption property of 2D VP nanosheets. Furthermore, the passively Q-switched Nd:YVO_4_ laser was realized with the 2D VP nanosheet-based SA, in which the average output power of 700 mW and the pulse duration of 478 ns were obtained. These results effectively reveal the nonlinear optical absorption characteristics of VP nanosheets, demonstrating their outstanding light-manipulating capabilities and providing a basis for the applications of ultrafast optical devices. Our results verify the excellent saturable absorption properties of 2D VP, paving the way for its applications in pulsed laser generation.

## 1. Introduction

Since the first discovery of graphene in 2004, atomically thin two-dimensional (2D) structural materials have become a research hotspot due to their unique optoelectronic properties, establishing great advantages in the construction of photonic and optoelectronic devices [1,2,3,4,5]. With the merits of broadband absorption [6,7,8,9], ultrafast carrier dynamic processing [10,11], excellent nonlinear optical response [12], easy parameter regulation [13], easy fabrication and intergradation [14], etc., 2D materials have been widely applied as saturable absorbers (SAs) to generate pulsed lasers [15,16,17], greatly facilitating the development of ultrafast lasers [18]. Since then, investigating novel 2D material-based SAs with excellent nonlinear optical performances is still in progress and has received widespread attention in the fields of materials and lasers [19,20].

Two-dimensional Violet phosphorus (VP), a novel allotrope of phosphorus, was first synthesized by Zhang et al. using the chemical vapor transport (CVT) method in 2019 [21]. This paved the way for in-depth exploration of the unique properties of 2D VP and broadened its applications, showing exciting research prospects in photonic and optoelectronic devices [22,23]. Compared with 2D black phosphorus (BP), 2D VP has higher carrier mobility, wide tunable bandgap, and significantly higher oxidation resistance. Schusteritsch et al. calculated anisotropic hole mobility with an upper bound between 3000 and 7000 cm^2^ V^−1^s^−1^ [24]. Moreover, compared with traditional black phosphorus (BP), Fali et al. reported the chemical degradation of exfoliated VP in comparison to BP under ambient conditions, indicating that VP exhibited a noticeably different and slower degradation process, which was beneficial for maintaining the long-term performance of devices [25]. Furthermore, the optical properties of 2D VP can be easily and precisely modulated by isoelectronic doping [26], strain [27], plasma treatment [28], and construct van der Waals heterojunction [29], etc. The third-order nonlinear optical response and saturable absorption properties play significant roles in optoelectronic and photonic applications. The challenges to measuring third-order nonlinear optical response and saturable absorption application in solid-state lasers arise from two main aspects: material selection; and material preparation. Firstly, the selected material should have a high nonlinear optical coefficient and saturation absorption, as well as great optical and environmental stability. Secondly, researchers need to prepare samples with large size, high quality, and high reproducibility for solid-state laser applications. As a new type of 2D material synthesized by the CVT method a few years ago, VP nanosheets have not been extensively researched, and it is difficult to exfoliate them into high-quality nanometer-scale sheets. Consequently, the ultrafast carrier dynamics, third-order nonlinear optical response, and saturable absorption properties of 2D VP nanosheets have been rarely studied, especially the applications in saturable absorption for solid-state lasers.

In this paper, the high-quality 2D VP nanosheet-based SA (2D VP-SA) was prepared using the liquid-phase exfoliation (LPE) technique. The ultrafast carrier dynamics and nonlinear optical properties were then investigated by using the pump–probe, open aperture Z-scan, and I-scan methods, showing the tremendous ultrafast carrier relaxation and strong nonlinear saturable absorption responses. Moreover, for the first time to our knowledge, a passive Q-switched (PQS) Nd:YVO_4_ laser operating at 1.06 μm was realized by using the as-prepared 2D VP-SA, generating a minimum pulse width of 478 ns and repetition frequency up to 400 kHz. The results indicate that 2D VP nanosheets have great potential for application in the field of pulsed laser generations.

## 2. Experimental Section

**Figure 1:**
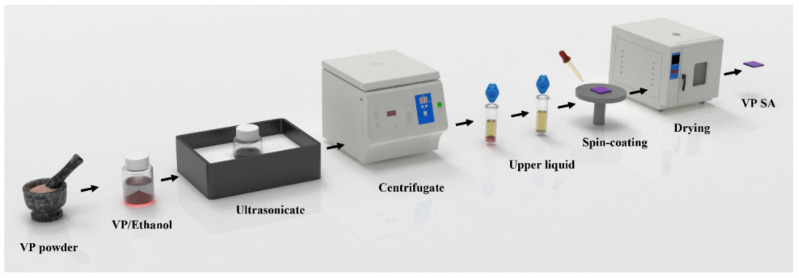
Schematic diagram of the process for preparing 2D VP-SA by LPE method.

As shown in Figure 1, the sample preparation process of 2D VP nanosheets was carried out by the LPE method [30,31,32]. Firstly, VP single crystals with 99.99% purity were ground and pestled into powder using an agate mortar to facilitate subsequent ultrasonic exfoliation. Secondly, 2 mg of fine powder was dispersed into 20 mL of anhydrous ethanol to prepare a mixed turbid solution, which was placed in a water bath sonicator for 6 h of ultrasonication under 300 W power. The bulk materials broke the van der Waals force between the layers and were ultrasonically exfoliated to form nanosheets. It should be noted that during the ultrasonic treatment process, the water temperature needs to be controlled below 25 °C using ice packs to prevent the VP nanosheets from oxidizing. During preparation, it was necessary to constantly replace the ice packs to maintain a low temperature. After that, the ultrasonicated solution was centrifuged for 15 min at a speed of 3000 rpm so that the large pieces of materials were deposited at the bottom of the solution, the thin layers of the sample were dispersed in the upper liquid, and then, the supernatant was collected for the next-step application. Finally, the treated supernatant was uniformly spin-coated on the sapphire substrate using a spin-coater with a rotational speed of 600 rpm. The sample was then placed in a vacuum drying oven at 45 °C for 2 h. The sample was prepared thoroughly.

## 3. Results and Discussion

**Figure 2:**
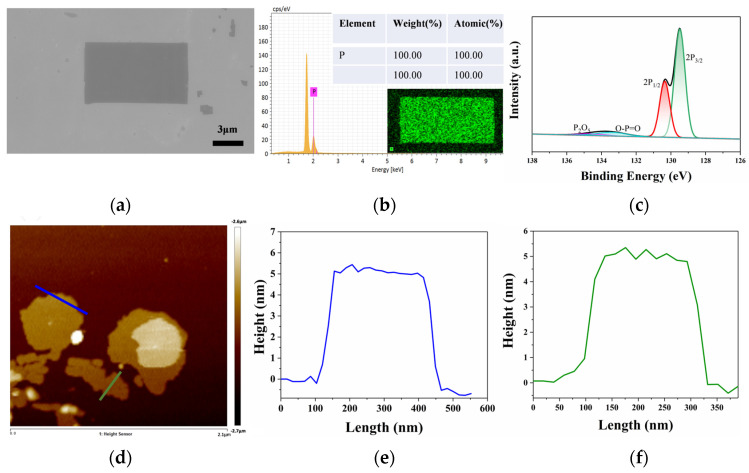
SEM, XPS, and AFM measurements of VP nanosheets. (**a**) SEM image of VP nanosheets with 3 μm scale. (**b**) EDX spectrum and the corresponding atomic ratio taken from the selected area. (**c**) The P 2p XPS spectra of VP nanosheets (**d**–**f**) AFM image and the typical height profiles of the VP nanosheets.

The comprehensive characterizations were performed on the as-prepared 2D VP nanosheets to determine their morphology, structure, and properties. Figure 2 shows the surface morphologies and structure images obtained from scanning electron microscopy (SEM) and atomic force microscopy (AFM), respectively. Figure 2a presents the lamellar structural morphology of 2D VP flakes by using SEM (G300 FE-SEM System, Carl Zeiss, Jena, Germany), indicating the high quality of the sample, and the other location of the sample is shown in Figure S1. During the preparation process of the samples, to eliminate the possibility of nanosheets being oxidized and confirm the elemental composition of the prepared materials, we performed the energy-dispersive X-ray spectroscopy (EDX) spectroscopy tests on the prepared 2D VP samples, and the results are shown in Figure 2b. The insert shows the EDX mapping image, where the shade of color indicates the distribution of elements in the 2D VP nanosheets. Using Silicon wafers as the substrate to exclude the interference of the O element, it can be seen that the sample can only detect the P element peak in addition to the Si peak of the substrate, indicating that the sample is not oxidized and exhibits high purity. The EDX measurement may not be accurate enough for elemental oxygen, so an X-ray Photoelectron Spectroscopy (XPS) test is needed for a more precise analysis. The spectra obtained using XPS (Thermo Fisher ESCALAB XI+, Waltham, MA, USA) are shown below. The 2p spectral peaks with binding energies of 129.6 eV and 130.5 eV correspond to 2p_1/2_ and 2p_3/2_, respectively, of the P=P bonds of the VP crystalline. Additionally, a peak at 133.8 eV can be observed, which can be deconvoluted into two peaks with binding energies of 133.4 eV and 134.5 eV, corresponding to O-P=O and P_2_O_5_, respectively [33]. It is worth noting that the peak analysis integrals show that the intensity ratio of P_x_O_y_ to P=P in the XPS spectra of the VP nanosheets is 0.09, indicating a small percentage. The oxidation features are negligible compared to the P 2p_3/2_ and P 2p_1/2_ [34]. Therefore, it can be concluded that the sample was not significantly oxidized after the LPE method and still maintained a relatively good crystal structure state and elemental composition. To determine the thickness of the 2D VP nanosheets, the samples were further tested and characterized using AFM (Bioscope Resolve, Bruker, Billerica, MA, USA). Figure 2c–e shows the AFM test results of the as-prepared 2D VP nanosheets, whose thickness was around 5 nm, and the number of layers of nanosheets was ~5 (the single layer thickness of ~1.1 nm [21]).

**Figure 3:**
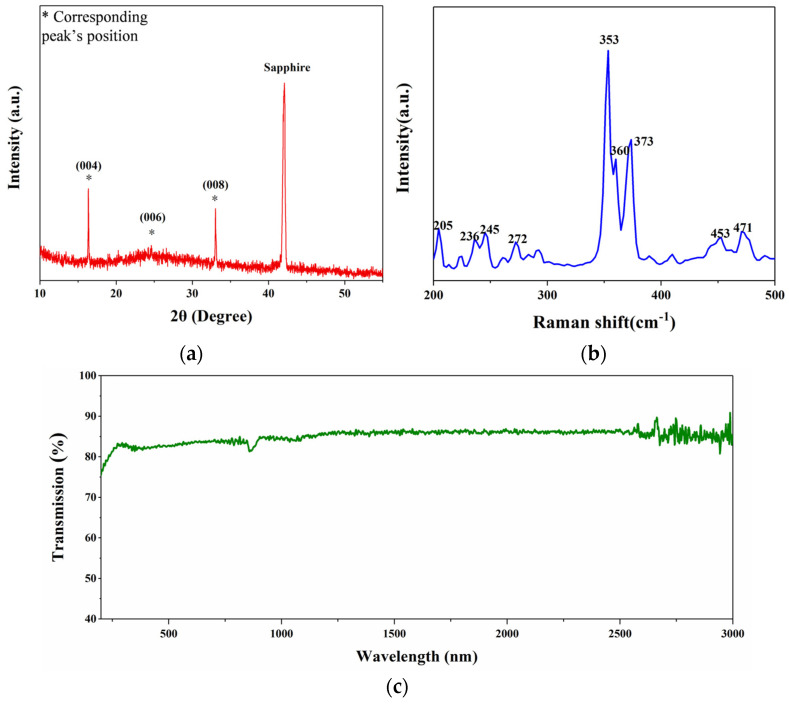
Characterization of VP nanosheets. (**a**) The X−ray diffraction (XRD) patterns; (**b**) Raman spectrum; (**c**) Linear transmission spectra of the prepared VP nanosheets.

The crystallinity and structure of the as-prepared VP nanosheets were further analyzed by X-ray diffraction (XRD) and Raman spectroscopy using an X-ray diffractometer (SmartLab 9KW, Rigaku, Tokyo, Japan) and a Raman spectrometer (PHS-3C, Horiba, Kyoto, Japan). The XRD spectrum of Figure 3a shows that the strong, sharp peaks with 2θ at 16.4°, 24.6°, and 33° correspond to (004), (006), and (008) reflections, respectively [21]. The sharp diffraction peaks indicate that the nanosheets have good crystallinity while further confirming the layered features of 2D VP nanosheets. The results indicate that the structure, good crystallinity, and high purity of 2D VP nanosheets were well-preserved after the LPE process. The Raman spectrum obtained by exciting the 2D VP nanosheets with a 473 nm laser is shown in Figure 3b. There are multiple characteristic peaks in the ranges of 200–300 cm^−1^ and 350–500 cm^−1^, which reflects the low symmetry of atomic vibrations. A large number of Raman patterns are attributed to the unique tubular structure of the VP crystals, which is consistent with the results of the previous report [35]. The characteristic peaks of the Raman spectrum in these two ranges are mainly caused by the stretching and bending bond angle distortion of the 2D VP layers. The medium vibration signals of P–P bonds are located at 205, 236, 245, and 272 cm^−1^, originating from the bond angle distortion. In addition, strong vibrational signals at high-frequency modes are also observed at 353, 360, 373, 453, and 471 cm^−1^, which are mainly caused by the stretching and bending of localized bonds [25,36]. The transmission spectrum measured by the UV-VIS-NIR spectrophotometer (UH4150, Hitachi, Tokyo, Japan) is shown in Figure 3c. The 2D VP nanosheets maintain good transmittance, showing broadband absorption from the UV to the NIR region, suggesting their potential application as SAs.

**Figure 4:**
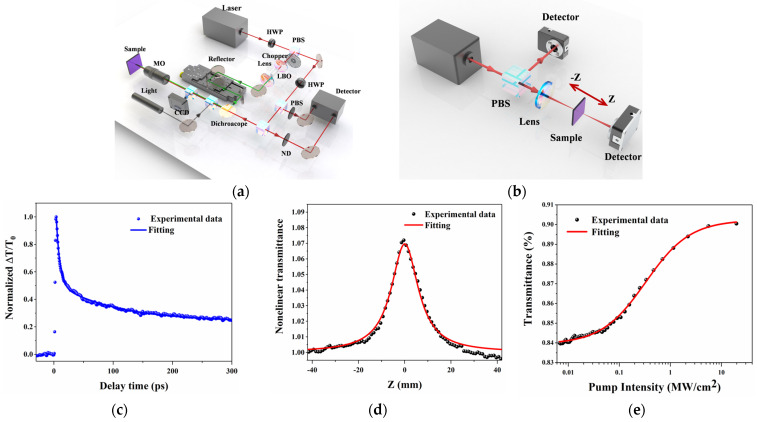
Nonlinear optical properties and ultrafast carrier dynamics of VP nanosheets. Experimental setup of (**a**) ultrafast pump−probe measurement and (**b**) Z−scan and I−scan measurements. (**c**) Normalized transmission of VP−SA with a probe laser at 1030 nm. (**d**) Z−scan and (**e**) I−scan measurements of the VP−SA at 1030 nm.

The pump–probe techniques, open aperture Z-scan, and I-scan were performed to characterize the ultrafast carrier dynamics and nonlinear optical response of the prepared 2D VP-SA. Ultrafast carrier dynamics in semiconductors play an important role in photonics and optoelectronics [37,38]. The schematic diagram of the nondegenerate pump–probe experimental setup is shown in Figure 4a. The same femtosecond laser source operating at 1030 nm as the Z-scan and the I-scan measurement was used. It was split into two paths: one was frequency doubled through an LBO crystal to generate a 515 nm laser to excite the sample, while the other was used as the probe laser to detect the pump-induced transmission variation in the sample. The pump and probe beams were both concentrated onto the sample using a 20× objective lens, and a CCD camera (Thorlabs, Newton, MA, USA, DCC1545M) was utilized to capture images of the sample and verify the coincidence of the pump and probe light centers. An adjustable gain-balanced photoreceiver (Newport, RI, USA, 2317NF) was used to detect changes in the probe caused by the pump. The chopper frequency was set at 2 kHz as a reference frequency for the lock-in amplifier, which was used to reduce the signal of the laser pulse repetition frequency to modulate the pump light and enhance the signal-to-noise ratio. Delay times on the order of femtoseconds were achieved by varying the optical path difference between the two beams of the laser by means of a stepper motor. As shown in Figure 4c, the transient optical response at the center wavelength of 1030 nm was detected by measuring the normalized transmittance (ΔT/T_0_) change in the 2D VP nanosheets. Here, T_0_ is the transmittance of the probe laser before pump laser excitation, and ΔT is the corresponding change in the probe laser after excitation. The positive ΔT/T_0_ signal illustrates the photobleaching characteristic owing to the excitation of hot carrier dynamics, which indicates that the 2D VP nanosheets exhibit a saturable absorption response at a wavelength of 1030 nm. In comparison, the same measurements were performed on a pure sapphire substrate, and no transient response was detected, suggesting that the dynamic response was entirely caused by the 2D VP nanosheets. For nondegenerate pump–probe measurements, the relaxation process can be fitted using a bi-exponential decay function:

(1) $$\frac{\Delta T}{T}=A1\;exp\left(-\frac{t}{\tau_{1}}\right)+A2\;exp\left(-\frac{t}{\tau_{2}}\right)$$

where *τ*_1_ and *τ*_2_ represent the fast and slow relaxation time associated with carrier-carrier intra-band and carrier-phonon inter-band scattering, respectively. A1 and A2 are their relative amplitudes. By using the Equation (1), *τ*_1_ and *τ*_2_ are determined to be 6.83 and 62.91 ps. The picosecond-scale ultrafast relaxation time of the 2D VP nanosheets suggests that it can be used for ultrafast optical signal devices.

A homemade open-aperture Z-scan device with the excitation laser source centered at 1030 nm was utilized, as shown in Figure 4b. The femtosecond laser source with a pulse width of 100 fs and a repetition rate of 100 MHz was split into two paths through a PBS, with one path directly incident to the detector D1 (S401C, Thorlabs, Newton, MA, USA), which served as a reference information to eliminate the instability of the laser source. The other path was focused on the sample through a convex lens, with f = 100 mm. After passing through the sample, it was received by a power meter of the same type, which recorded the change in laser intensity after passing through the sample as a function of position. The sample was placed on a stepper motor-controlled motorized displacement stage, which was controlled by a computer program to move back and forth near the focal point. Figure 4d shows the normalized transmittance with intensity dependence as a function of the position z. Under a certain incident laser energy, the transmittance gradually increased as the sample moved toward the beam waist position, and the transmittance curve presented an obvious wave crest at the focal point, indicating the typical saturable absorption characteristics of 2D VP nanosheets originating from the Pauli blocking effect. The Z-scan curves were fitted and analyzed using the following formulas:

(2) $$T=\sum_{m=0}^{\infty }\frac{{\left[-q_{o}\left(z,0\right)\right]}^{m}}{{\left(m+1\right)}^{\frac{3}{2}}},\left(m\in N\right),q_{0}\left(z,0\right)=\frac{\beta_{eff}\cdot L_{eff}\cdot I_{0}}{1+{\left(\frac{z}{z_{0}}\right)}^{2}}$$

Figure 4d shows the experimental data curves and the fitting results. The obtained values of the nonlinear absorption coefficients *β*_eff_ were determined to be ~−2.18 × 10^4^ cm/MW. In order to further analyze the saturable absorption properties of the 2D VP nanosheets, the I-scan technology was conducted, as shown in Figure 4e. The experimental setup of the I-scan device is similar to the Z-scan device, which places a variable attenuator in front of the laser while keeping the sample position fixed, changing the incident light intensity using an attenuator and exploring the relationship between pump intensity and transmittance. The parameters can be obtained by fitting the curves using the following formula:

(3) $$T=1-\frac{∆R}{1+\frac{I}{I_{S}}}-\alpha_{ns}$$

where the value of modulation depth Δ*R*, saturation intensity *I*_s_, and nonsaturable losses can be obtained as 6.3%, 329 kW/cm^2^, and 9.8%, respectively. The values of nonlinear absorption parameters of different materials are shown in Table 1. For passively Q-switched, recovery time plays a role but is not the only determining factor; low nonsaturable loss and large nonlinear absorption coefficient are also necessary. It is obvious that the 2D VP nanosheets have great nonlinear absorption properties, indicating that 2D VP nanosheets have great potential in applications as SAs for generating pulsed lasers.

**Figure 5:**
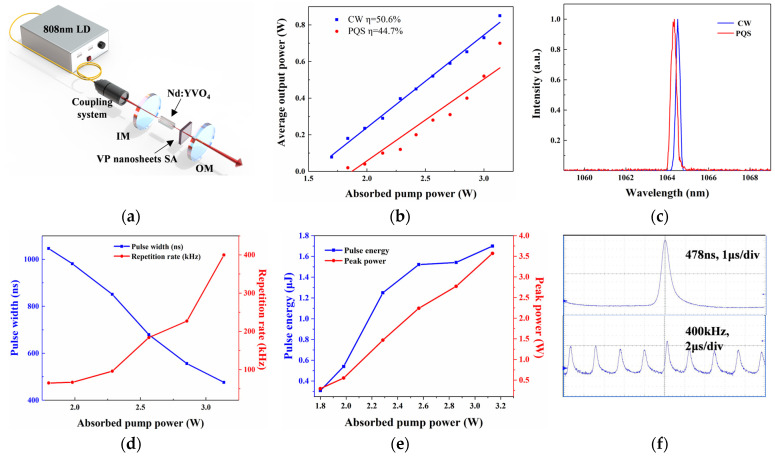
The Q-switched results of Nd:YVO_4_ based on 2D VP-SA. (**a**) Experimental setup of VP nanosheets-based PQS all-solid-state lasers. (**b**) The relationship between the CW and PQS laser output power and the absorbed pump power. (**c**) The CW and PQS operation emitted central wavelength. (**d**) Pulse widths and repetition rates versus absorbed pump powers. (**e**) Pulse energy and peak power versus absorbed pump powers. (**f**) The typical PQS pulse sequences and the single-pulse shape.

In order to realize the application of 2D VP nanosheets in all-solid-state lasers, the 2D VP-SA was inserted into a laser resonator to realize the passively Q-switched (PQS) laser pulses. The PQS experimental setup is shown in Figure 5a. The cavity length was 22 mm. An a-cut Nd:YVO_4_ crystal with a dimension of 3 × 3 × 4 mm^3^ was applied as the gain medium. A fiber-coupled laser diode with a central wavelength of 808 nm was used as the pump source, with a fiber core of 400 μm and a numerical aperture of 0.22. The pump source was focused into the crystal after a 1:1 optical focusing system. The input mirror was a flat mirror, while the output mirror was a flat mirror with a transmittance of 5% at 1064 nm. After inserting the 2D VP-SA into the cavity, the cavity mirror and incident pump power were precisely adjusted, and PQS was realized when the absorbed pump power reached 1.7 W. Figure 5b depicts a plot of the average output power and absorbed pump power for the continuous wave (CW) and PQS operations. With the increase in absorbed pumping power, the output power gradually increases, showing a linear relationship with the absorbed pumping power and the slope efficiency of 50.6% and 44.7%, respectively. The highest PQS average output power reached 700 mW when the absorbed pump power increased to 3.1 W. With the continued increase in pump power, the PQS pulse waveform becomes very unstable until it returns to the continuous laser. Figure 5c displays the PQS operation emitted at a central wavelength of 1064.4 nm at the highest average output power, while the CW operation emitted a wavelength of 1064.5nm. The blue shift occurred in the central wavelength, which could be led by the insertion loss of the 2D VP-SA in the resonator. The relationship of the pulse width and repetition rate in contrast with the absorbed pump power is shown in Figure 5d. As the increased absorption pump power, the pulse width gradually decreased from 1.05 μs to the minimum value of 478 ns, and the repetition frequency increased from 65 kHz to the maximum value of 400 kHz. According to Figure 5e, the corresponding highest output pulse peak power was 3.57 W, and the single pulse energy was 1.7 μJ. Figure 5f shows the characteristic pulse train and the shortest pulse profile under the maximum output power. The output performance of PQS suggests that 2D VP nanosheets are a great candidate as SA material for producing laser pulses and other optoelectronic devices.

## 4. Conclusions

In summary, based on the high-quality 2D VP nanosheets SA prepared by the LPE method, the nonlinear optical absorption properties and ultrafast carrier dynamics were studied using pump–probe, Z-scan, and I-scan techniques, demonstrating significant saturable absorption responses. The nonlinear absorption coefficient βeff, the saturation intensity Is, the modulation depth ΔR, and the nonsaturable loss were determined to be −2.18 × 10^4^ cm/MW, 329 kW/cm^2^, 6.3%, and 9.8%, respectively. The intra- and inter-band ultrafast relaxation times of 2D VP nanosheets were ~6.83 ps and ~62.91 ps with a probe laser operating at 1.03 μm. In addition, the prepared 2D VP nanosheets were used in a PQS Nd:YVO_4_ solid-state laser operating at 1064 nm, generating a pulse laser with a pulse width of 478 ns and a repetition rate of 400 kHz. This work shows that 2D VP nanosheets are excellent SA materials that can be used as great nonlinear optical modulation materials for pulse modulation of solid-state lasers.

## Figures and Tables

**Table 1:** Summarization of nonlinear absorption parameters of different materials.

Materials	τ_1_ (ps)	τ_2_ (ps)	β_eff_ (cm/GW)	$\alpha_{ns}$ (%)	Ref.
Graphene	0.13	3.75	−1.2 × 10^1^	/	[39]
Graphene	/	/	/	10.95	[40]
Graphene/MoS_2_	0.6	5	/	13.1	[41]
WS_2_	16.7	166.7	3.1 × 10^−4^	/	[42]
MoSe_2_	2.16	210.13	−1.7 × 10^−2^	44.8	[43]
Ti_2_CT_x_ MXene	/	/	/	23.1	[44]
VP	6.83	62.91	−2.18 × 10^7^	9.8	This work

## Data Availability

The raw data supporting the conclusions of this article will be made available by the authors on request.

## Supplementary Materials

The following supporting information can be downloaded at https://www.mdpi.com/article/10.3390/ma17112585/s1, Figure S1: The other location/different scale of SEM.
