# Supplementary figures and images for: Nonlinear Optical Saturable Absorption Properties of 2D VP Nanosheets and Application as SA in a Passively Q-Switched Nd:YVO4 Laser
Source: Materials (Basel). 2024 May 28;17(11):2585. doi: 10.3390/ma17112585 (PMC11174069; doi:10.3390/ma17112585)

## Supplementary Materials

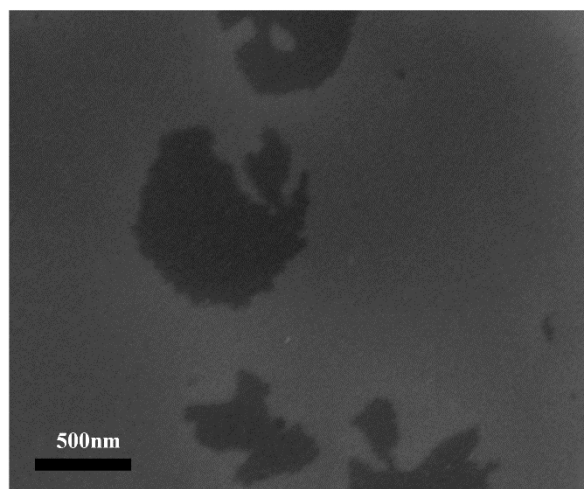

Figure S1. The other location/ different scale of SEM

Supplement: Supplementary file 1 [file materials-17-02585-s001.zip › materials-2994482-supplementary.pdf]
